# The high expression of ADRM1 in hepatocellular carcinoma is closely related to tumor immune infiltration and is regulated by miR-891a-5p

**DOI:** 10.1038/s41598-024-64928-2

**Published:** 2024-06-18

**Authors:** Ziqi Shao, Yuan Wang, Yuejun He, Chen Zhang, Yandong Zhao, Mimi Zhang, Qiang Li, Jian Wang

**Affiliations:** Department of General Surgery of the Second Affiliated Hospital of Xuzhou Medical University, No.32 of Meijian Road, Xuzhou, 221000 Jiangsu Province China

**Keywords:** Cancer, Cell growth, Cell migration, Immune evasion

## Abstract

Liver cancer is one of the most common malignant tumors worldwide. Although some progress has been made in the diagnosis and treatment of Hepatocellular carcinoma (HCC), the diagnosis and treatment of HCC is still facing great challenges because of the high mortality rate and poor prognosis of HCC. The purpose of this study was to explore the relationship between adhesion-regulating molecule1 (ADRM1), and liver cancer, and the relationship between prognoses. ADRM1 is highly expressed in tumors and is closely associated with the prognosis of patients with liver cancer. In our previous study, we found that ADRM1 was highly expressed in HCC and was closely related to tumor immune and immune checkpoint levels in HCC. We validated the immune expression of ADRM1 in liver cancer cells using flow cytometry. In hepatocellular carcinoma tissues, miR-891a-5p regulates ADRM1. Upregulation of miR-891a-5p upregulates ADRM1, and downregulation of miR-891a-5p downregulates ADRM1. It is suggested that ADRM1 plays a key role in the occurrence and development of hepatocellular carcinoma. This study is expected to provide new ideas for the research and development of anti-HCC drugs targeting miR-891a-5p/ADRM1. However, further trials are needed to confirm these results and explore the actual results in patients with HCC.

## Introduction

HCC is one of the most common malignant tumors in the world, ranking fourth in the world. It accounts for an alarming two million deaths annually, contributing to 4% of all global fatalities (1 out of every 25 deaths)^1–3^. HCC has a complex origin and involves various factors, such as viral infections, cirrhosis, excessive alcohol consumption, and immune system dysregulation playing a role^4–6^. Despite advancements in HCC diagnosis and treatment, significant challenges remain in its high mortality rate and unfavorable prognosis, primarily attributed to frequent recurrences and the spread of cancer to other parts of the body^7^. Liver cancer is mostly moderate and severe and is mainly treated by surgery at present, but there is no effective clinical method so far^8,9^. Liver cancer is one of the most common malignant tumors in China; therefore, there is an urgent need to identify a new mechanism for the occurrence and development of liver cancer.

ADRM1 is a key molecule in the binding of 26s to its substrate and is a connexin of 26s^10,11^. Previous studies have shown that ADRM1 regulates cell differentiation, migration, and adhesion. ADRM1 is a oncogenic factor that plays an important role in cancer^12–17^. ADRM1 is highly expressed in ovarian cancer cells and is closely associated with poor patient prognosis^13^. In addition, ADRM1 plays an important role in tumorigenesis and development^15^. Moreover, the transcription of ADRM1 has been shown to stimulate the growth of colorectal cancer^16^. In 2020, Liang reported elevated levels of ADRM1 expression is indicative of an unfavorable prognosis and can be suppressed by RA190 in HCC^19^. However, this study focused on the association between miRNA891a-5p and the expression, prognosis, and molecular mechanism of action of ADRM1 in HCC and its relationship with immune infiltration of HCC remain unclear. Based on the ADRM1 gene, this study analyzed the relationship between ADRM1 expression in many types of tumor tissues and the prognosis of tumor patients. Based on this, we studied the relationship between the expression of ADRM1 in hepatocellular carcinoma and immune cell infiltration, immune cell labeling, and immune test sites. Simultaneously, through the detection of ADRM1 upstream of miRNAs in hepatocellular carcinoma, its function can be regulated. The results showed a notable association between ADRM1 expression and adverse prognosis along with heightened immune infiltration of tumors in patients with HCC. Additionally, we identified miRNA-891a-5p as a crucial regulator that specifically targets ADRM1 in HCC cells.

## Results

### Pan-cancer displays the manifestation of ADRM1

**Figure 1:**
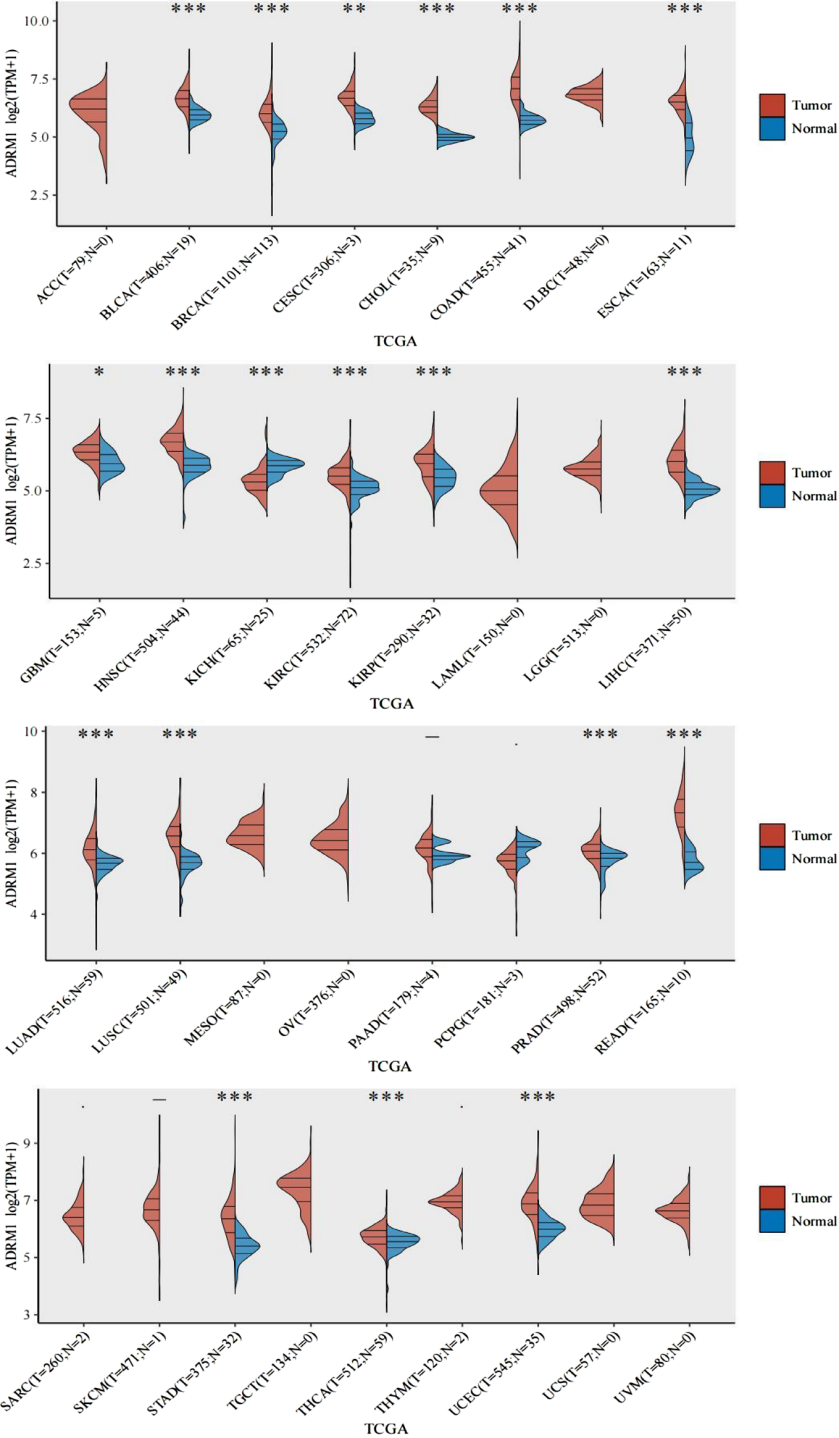
(**A**) ADRM1 expression in various malignancies: The expression levels of ADRM1 were analyzed in 32 different types of human cancers using TCGA datasets, including both cancerous and normal tissues. *P < 0.05, **P < 0.01, ***P < 0.001.

Therefore, we aimed to further understand the function of ADRM1 in tumors. We detected ADRM1 protein in 32 types of tumors. The results showed that ADRM1 protein was significantly up-regulated in 18 kinds of tumors, including Bladder Urothelial Carcinoma (BLCA), Breast invasive carcinoma (BRCA), Cervical squamous cell carcinoma (CESC), Cholangial carcinoma (CHOL), Colon adenocarcinoma (COAD), Esophageal carcinoma (ESCA), Glioblastoma multiforme (GBM), Head and Neck squamous cell carcinoma (HNSC), Kidney renal clear cell carcinoma (KIRC), Kidney renal papillary cell carcinoma (KIRP), Liver hepatocellular carcinoma (LIHC), Lung adenocarcinoma (LUAD), Lung squamous cell carcinoma (LUSC), Prostate adenocarcinoma (PRAD), Rectum adenocarcinoma (READ), Stomach adenocarcinoma (STAD), Thyroid carcinoma (THCA), and Uterine Corpus Endometrial Carcinoma (UCEC). After Kidney Chromophobe (KICH) (Fig. 1), the expression of ADRM1 decreased significantly. There was no significant difference in the expression of ADRM1 between Pancreatic adenocarcinoma (PAAD) group and PCPG group (Fig. S1A). To confirm our results, we conducted an extensive examination utilizing the TCGA + GTEx repository, confirming a notable increase in ADRM1 in BLCA, CESC, COAD, ESCA, GBM, KIRC, KIRP, LIHC, LUAD, LUSC, READ, STAD, and UCEC compared to their respective healthy controls (Fig. S1B–N). In contrast, we observed a notable decrease in ADRM1 expression in THCA (Fig. S1O). There was no notable variation in the expression of ADRM1 in BRCA (Fig. S1P), PRAD (Fig. S1Q), or KICH (Fig. S1R). Unfortunately, because of the lack of GTX data, evaluation of ADRM1 expression in CHOL and HNSC was not possible. Based on the above results, we believe that ADRM1 plays an important regulatory role in the above 13 types of tumors; however, its specific mechanism remains to be further elucidated to clarify its role in antitumor and potential applications.

### The predictive significance of ADRM1 in human malignancies

**Figure 2:**
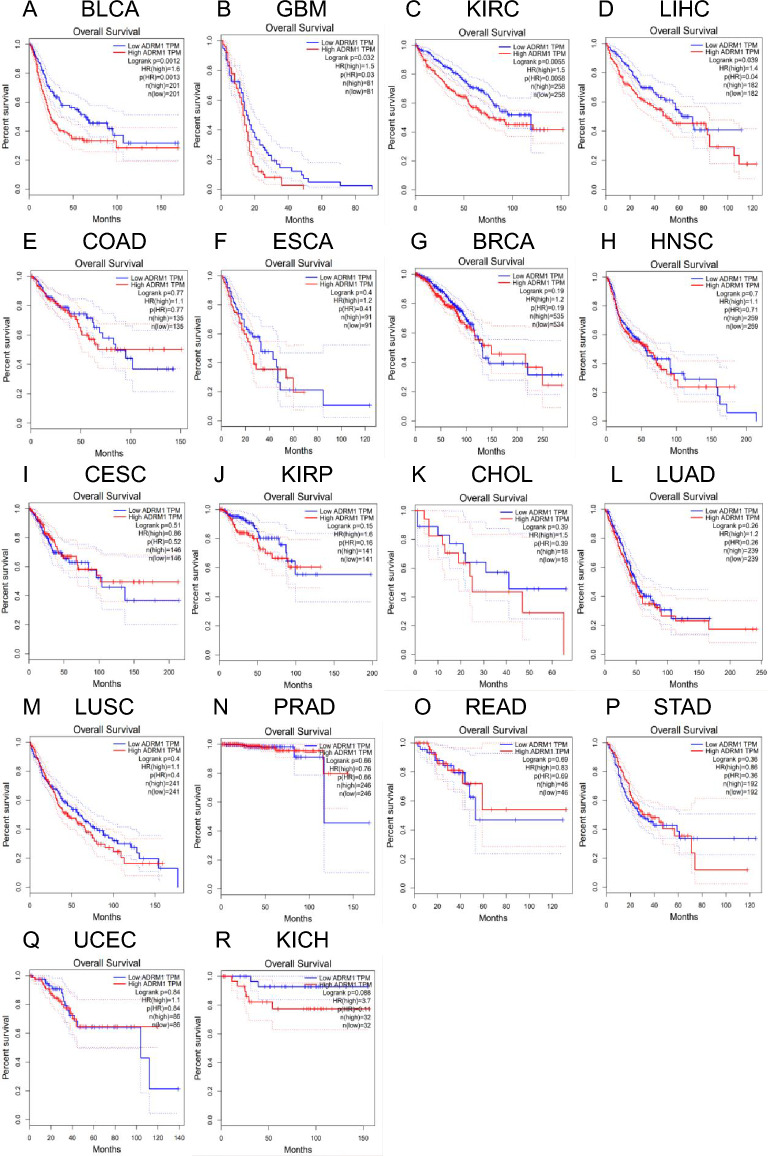
The GEPIA database analyzed the overall survival (OS) of ADRM1 in different types of human cancer. *P value < 0.05, **P value < 0.01, ***P value < 0.001.

The purpose of this project was to use GEPIA data to verify the ADRM1 gene expression profile and clarify the relationship between ADRM1 gene expression and tumorigenesis, development, and prognosis. BLCA, GBM, KIRC, LIHC, COAD, ESCA, BRCA, HNSC, CESC, KIRP, CHOL, LUAD, LUSC, PRAD, READ, STAD, UCEC, and KICH were collected. The results of our study suggest that increased ADRM1 expression in BLCA, GBM, KIRC, and LIHC is linked to unfavorable overall survival rates (OS) (Fig. 2A–D). However, there was no significant correlation between ADRM1 and overall survival rates (OS) in COAD, ESCA, respectively, BRCA, HNSC, CESC, KIRP, CHOL, LUAD, LUSC, PRAD, READ, STAD, UCEC, and KICH (Fig. 2E–R). Furthermore, an unfavorable prognosis was associated with high ADRM1 expression in BLCA, BRCA, KIRC, KIRP, and LIHC for disease-free survival (RFS) (Fig. S2A–E). Our previous work suggested that ADRM1 has potential clinical value in HCC; however, its role in HCC remains unclear.

### The expression of ADRM1 in HCC and the correlation between ADRM1 and the clinicopathological characteristics of HCC patients

**Figure 3:**
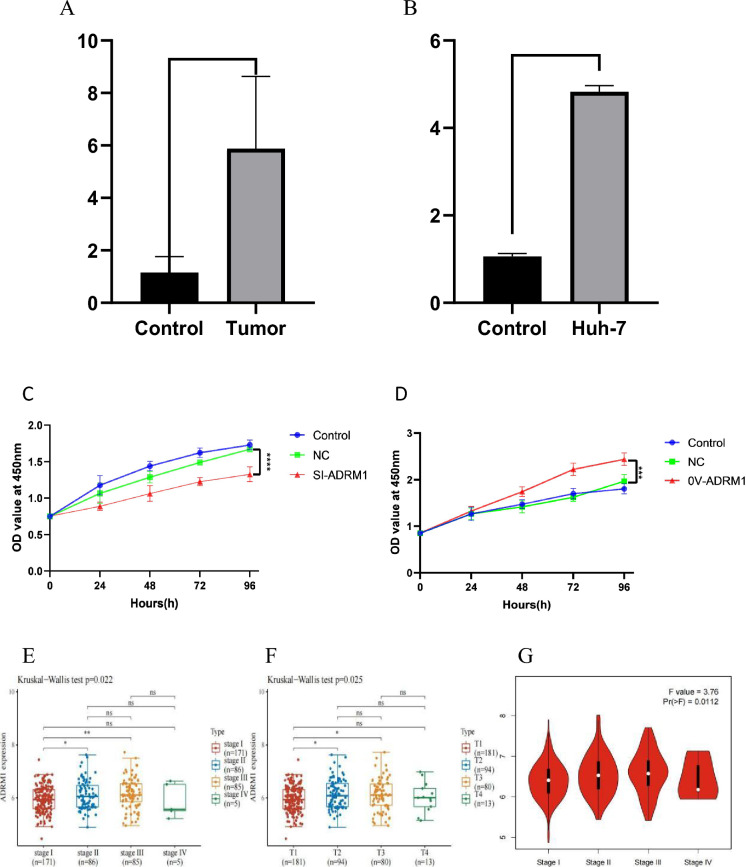
(**A**) The ADRM1 expression in paired HCC tissues using qTR-PCR. (**B**) The ADRM1 expression in Huh-7using qTR-PCR. (**C**, **D**) Cell viability after ADRM1 inhibition or upregulation assessed by CCK8 assay. (**E**) ADRM1 protein in LIHC and its correlation with clinical and pathological indexes. (**F**) Expression of ADRM1 gene in breast cancer cells and paracancerous tissues. (**G**) The expression of ADRM1 in LIHC (GEPIA) is closely related to the different stages of cancer.

**Figure 4:**
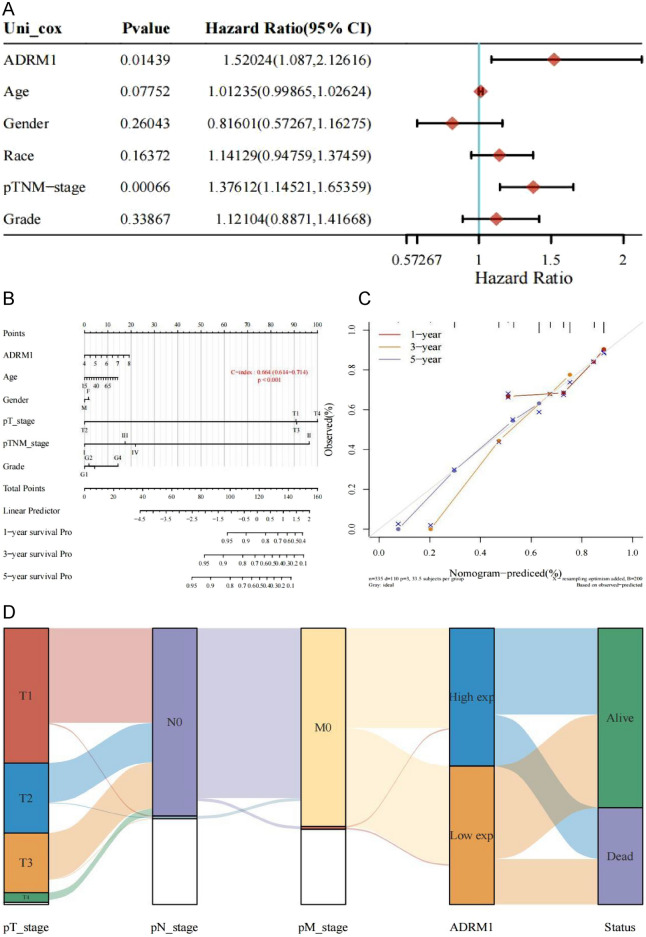
(**A**) Hazard ratio and *P*-value of constituents involved in the univariable Cox regression. (**B**) Nomogram consisting of risk score and other clinical indicators to predict the 1-year, 3-year and 5-year OS of the patients with HCC. (**C**) calibrate the overall survival nomogram of the subjects after treatment as contemporary. (**D**) Sankey diagram.

To investigate the role of ADRM1 in HCC, we examined the expression of ADRM1 in HCC tissue and Huh-7cell, and tested the relationship between ADRM1 levels and tumor stages and grades of HCC patients. We performed qRT-PCR in Huh-7cell line and HCC tissue to validate the expression of ADRM1. The findings indicate that ADRM1 is significantly upregulated in HCC tissue (Fig. 3A) and Huh-7 cell line (Fig. 3B). Subsequently, CCK8 analysis was performed to examine the effect ofADRM1 on HCC cell proliferation. The results demonstrated that overexpression of ADRM1 accelerated the proliferation of HCC cells (Fig. 3C), whereas knockdown of ADRM1 inhibited HCC cell proliferation (Fig. 3D). As depicted in Fig. 3E–G, the expression of ADRM1 was found to be significantly associated with various grade stages (Fig. 3E, *P* = 0.022) and T stage (Fig. 3F,  *P* = 0.025). However, no significant correlation was observed between grade and T stages. This was confirmed using the GEIPIA database (Fig. 3G). Analysis of a single variable showed that ADRM1 (HR = 1.52, *P* = 0.014), pT-stage (HR = 1.67, *P* < 0.0001), and pTNM-stage (HR = 1.38, *P* = 0.000) had prognostic significance for the overall survival of HCC (Fig. 4).

### The expression of ADRM1 positively correlates with immune cell infiltration in HCC

**Figure 5:**
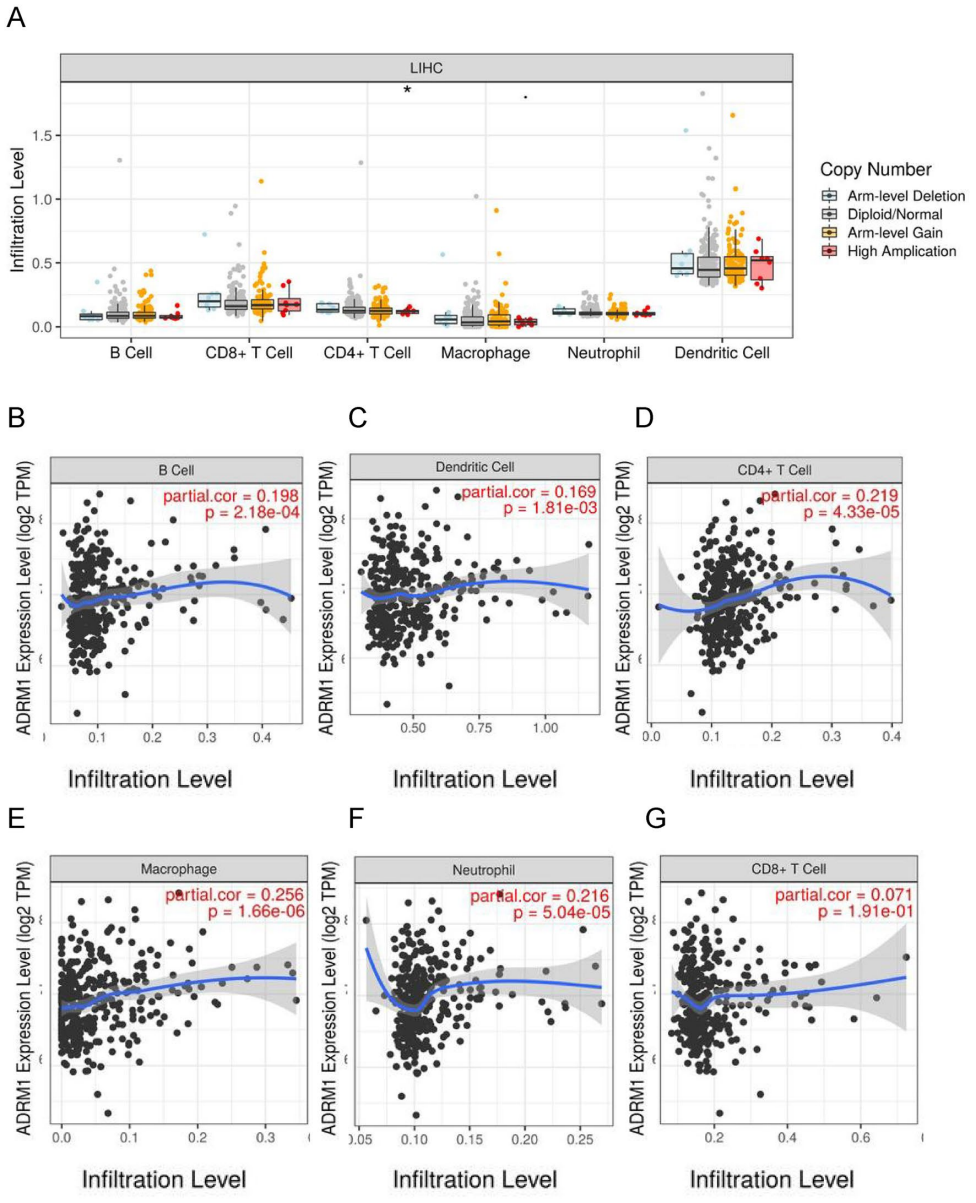
The association of immune cell infiltration with ADRM1 level in HCC. (**A**) The infiltration level of various immune cells under different copy numbers of  B cell (**B**), Dendritic cell (**C**), CD4^+^T cell (**D**), Macrophage (**E**), Neutrophil (**F**), CD8^+^T cell (**G**) infiltration level in HCC.

To investigate the cause of the negative prognosis associated with increased ADRM1 expression, we utilized TIMER to uncover the relationship between ADRM1 and the levels of six infiltrating immune cell subtypes: B cells, CD8^+^ T cells, CD4^+^ T cells, macrophages, neutrophils, and dendritic cells. In LIHC, the permeability of immune cells did not change significantly because of the number of ADRM1 replicates (as shown in Fig. 5A). Figure 5B–F shows that there is a significant correlation between ADRM1 and B lymphocytes, CD4^+^T lymphocytes, macrophages, neutrophils, and dendritic cells (*P* < 0.001). However, no significant correlation was observed between ADRM1 expression and CD8 ^+^T cells (Fig. 5G, *P* > 0.05).

### The relationship between ADRM1 and immune cell biomarkers in HCC

**Figure 6:**
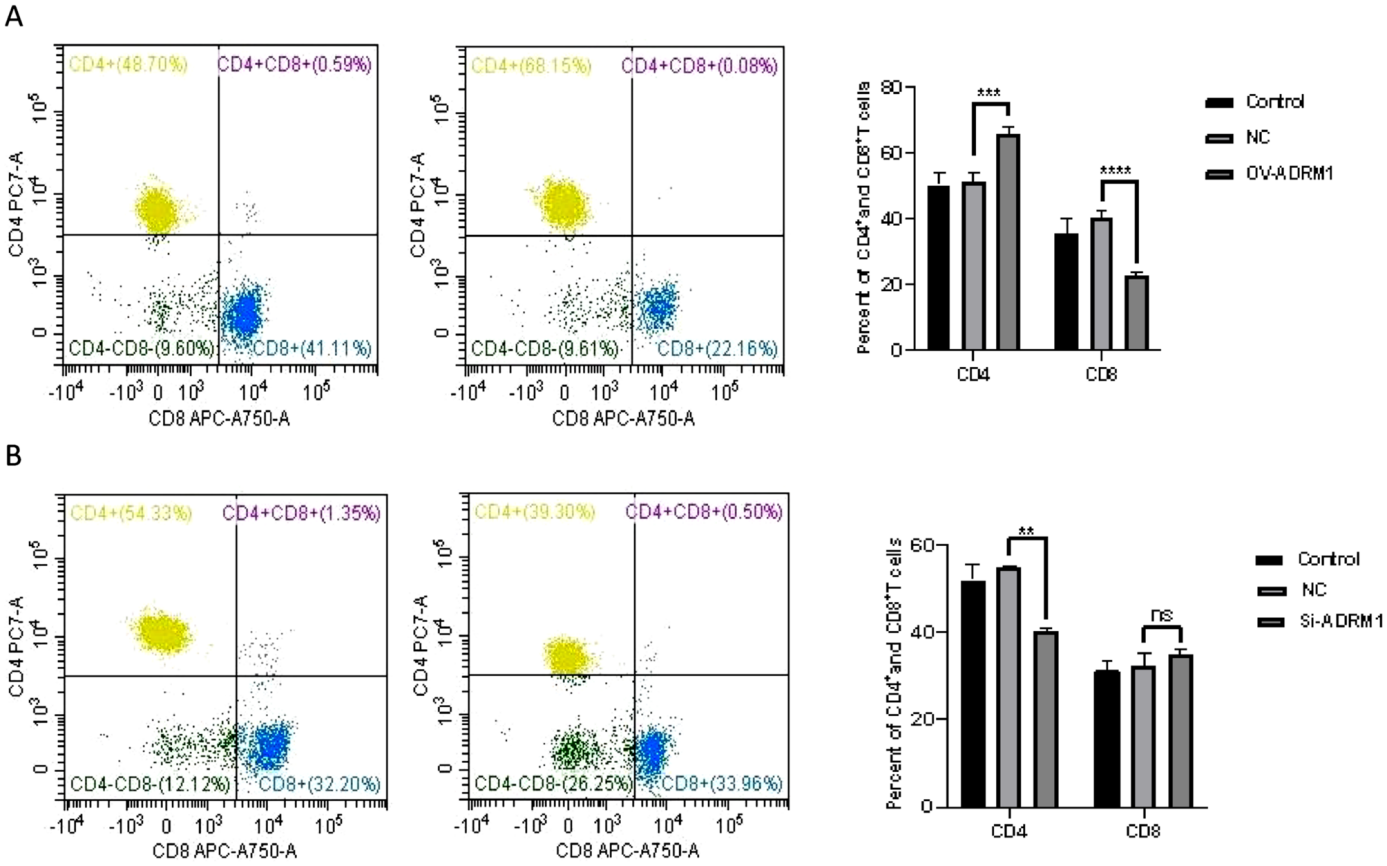
(**A**) Percentage and number of CD4^+^T cells and CD8^+^T cells in OV-ADRM1 Huh-7. (**B**) Percentage and number of CD4^+^T cells and CD8^+^T cells in Si-ADRM1 Huh-7.

We used R software to analyze the correlation between ADRM1 and immune cell biomarkers in liver cancer. Additional studies have shown that ADRM1 expression correlates well with various immune cell markers in liver cancer tissues. In our pre-experiment, we found that ADRM1 was highly expressed in hepatocellular carcinoma and positively correlated with B cell biomarkers (CD19), CD8^+^T cell biomarkers (CD8A and CD8B), M1 macrophage biomarker (IRF5), M2 macrophage biomarker (VSIG4), Neutrophil biomarkers (CEACAM8 and ITGAM), and Dendritic cell biomarkers (HLA-DPB1, HLA-DRA, HLA-DPA1, and ITGAX) within the HCC context (Table1). These findings were partially consistent with the notion that ADRM1 positively affects immune cell infiltration. Based on the aforementioned experimental findings, we conducted supplementary investigations to verify the correlation between the expression levels of ADRM1 and T immune cells using flow cytometry. We observed a notable increase in the percentage of CD4^+^T cells (65.85%, *P* = 0.0002, Fig. 6A), accompanied by a decrease in the percentage of CD8^+^T cells (22.89%, *P* < 0.0001) in ADRM1-overexpressing Huh-7 cells. Conversely, in ADRM1-knockdown Huh-7 cells, there was a significant reduction in CD4^+^T cells (40.28%, *P* = 0.001), whereas no discernible difference was observed in CD8^+^T cells (35.14%, *P* = 0.648, Fig. 6B).

**Table 1:** Correlation analysis between ADRM1 and biomarkers of immune cells in HCC determined by TCGA database and R software package.

Immune cell	Biomarker	R value	*P* value
B cell	CD19	0.21^a^	4.08E−05***^a^
	CD79A	0.07	0.207
	CD8A	0 11^a^	0 031*^a^
CD8^+^T cell	CD8B	0. 12^a^	0.02*^a^
CD4^+^T cell	CD4	0.07	0.171
	NOS2	− 0.02	0.756
M1 macrophage	IRF5	0.38^a^	1.70E−14***^a^
	PTGS2	0.00	0.982
	CD163	0.06	0.259
M2 macrophage	VSIG4	0. 14^a^	0.007**^a^
	MS4A4A	0.08	0.118
	CEACAM8	0.10^a^	0.044*^a^
Neutrophil	ITGAM	0.22^a^	1.90E−05***^a^
Dendritic cell	CCR7	0.00	0.982
	HLA-DPB1	0.18^a^	0.000***^a^
	HLA-DQB1	0. 14^a^	0.007**^a^
	HLA-DRA	0.13^a^	0.016*^a^
	HLA-DPA1	0.10^a^	0.049*^a^
	CD1C	0.04	0.400
	NRP1	0.10	0.060
	ITGAX	0.17^a^	0.001***^a^

^a^These results are statistically significant.

*P value < 0.05, **P value < 0.01, ***P value < 0.001.

### The correlation between ADRM1 and immune checkpoints in HCC

**Figure 7:**
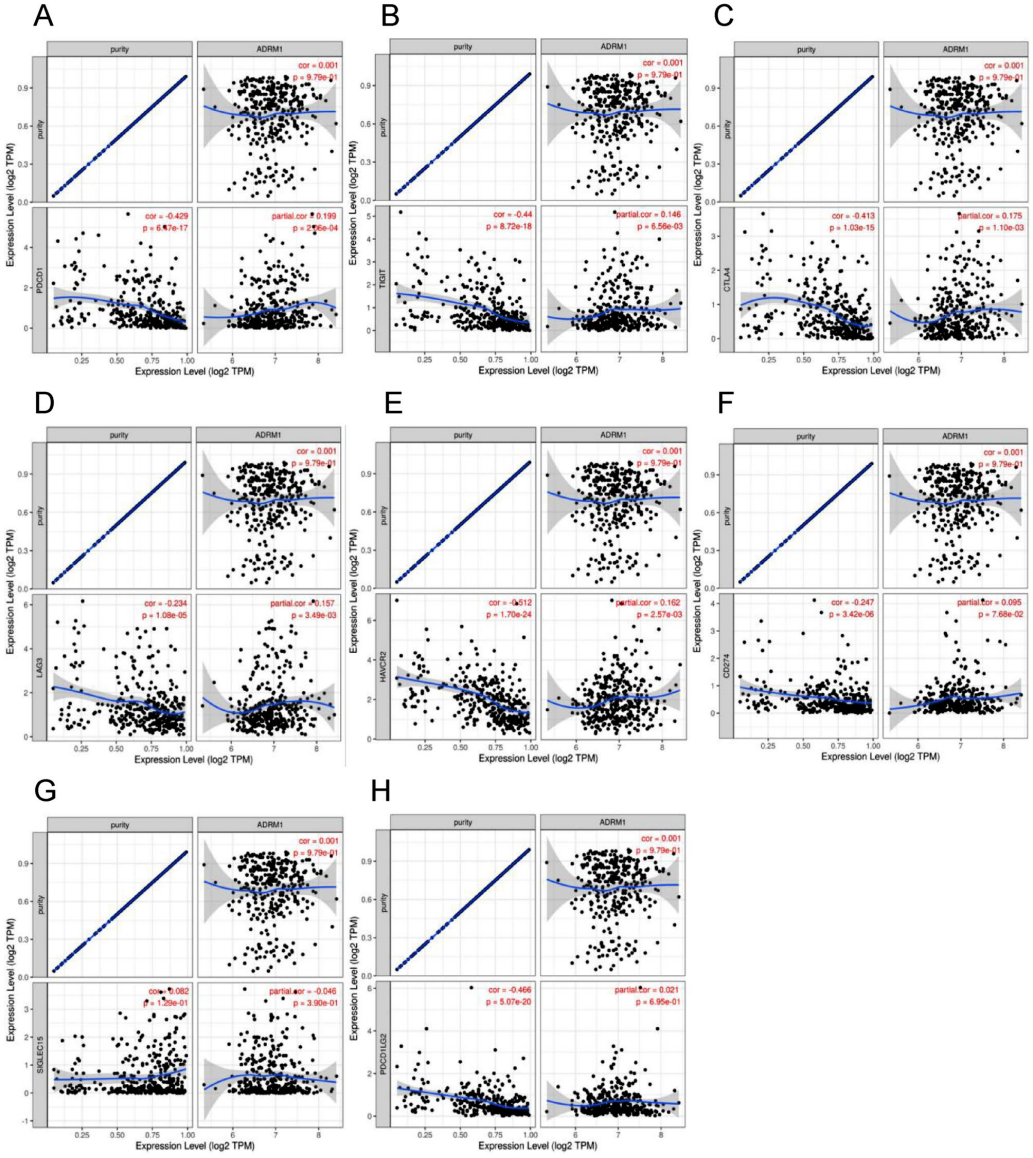
Spearman correlation of ADRM1 expression with PDCD1 (**A**), TIGIT (**B**), CTLA4 (**C**), LAG3 (**D**), HAVCR2 (**E**), CD274 (**F**), SIGLEC15 (**G**), and PDCD1LG2 (**H**) expression in HCC adjusted by purity using TIMER.

Given the potential oncogenic function of ADRM1 in hepatocellular carcinoma, our objective was to investigate its correlation with vital immune checkpoints that contribute to the evasion of tumor immunity. Therefore, we used the TIMER database to detect the correlation between ADRM1 and the immune checkpoints PDCD1, PDCD1LG2, CD274, HAVCR2, LAG-3, TIGIT, SIGLEC15, and CTLA-4 in LIHC. This study found a correlation between PDCD1, TIGIT, CTLA4, LAG3, and HAVCR2 (Fig. 7A–E), but a correlation with CD274, SIGLEC15, and PDCD1LG2 (Fig. 7F–H) is not obvious.

Afterwards, we confirmed the correlation between ADRM1 and immune checkpoints in HCC by utilizing the GEPIA database. The expression of ADRM1 (Fig. S3A–H) was significantly linked to PDCD1, CTLA4, and HAVCR2. These findings emphasize the significance of PDCD1, CTLA4, and HAVCR2 as immune regulatory points of ADRM1, indicating that evasion of the immune system by tumors could have a crucial impact on the development of HCC through ADRM1.

### miRNA-891a-5p has the potential to target ADRM1-3′UTR functionally

In this part of the study, our objective was to investigate the possible regulation of ADRM1 by miRNAs in the upstream region and to confirm the predicted interaction through a dual-luciferase reporter gene assay. By leveraging the StarBase database, we identified miRNA-891a-5p as an upstream regulator of ADRM1.

**Figure 8:**
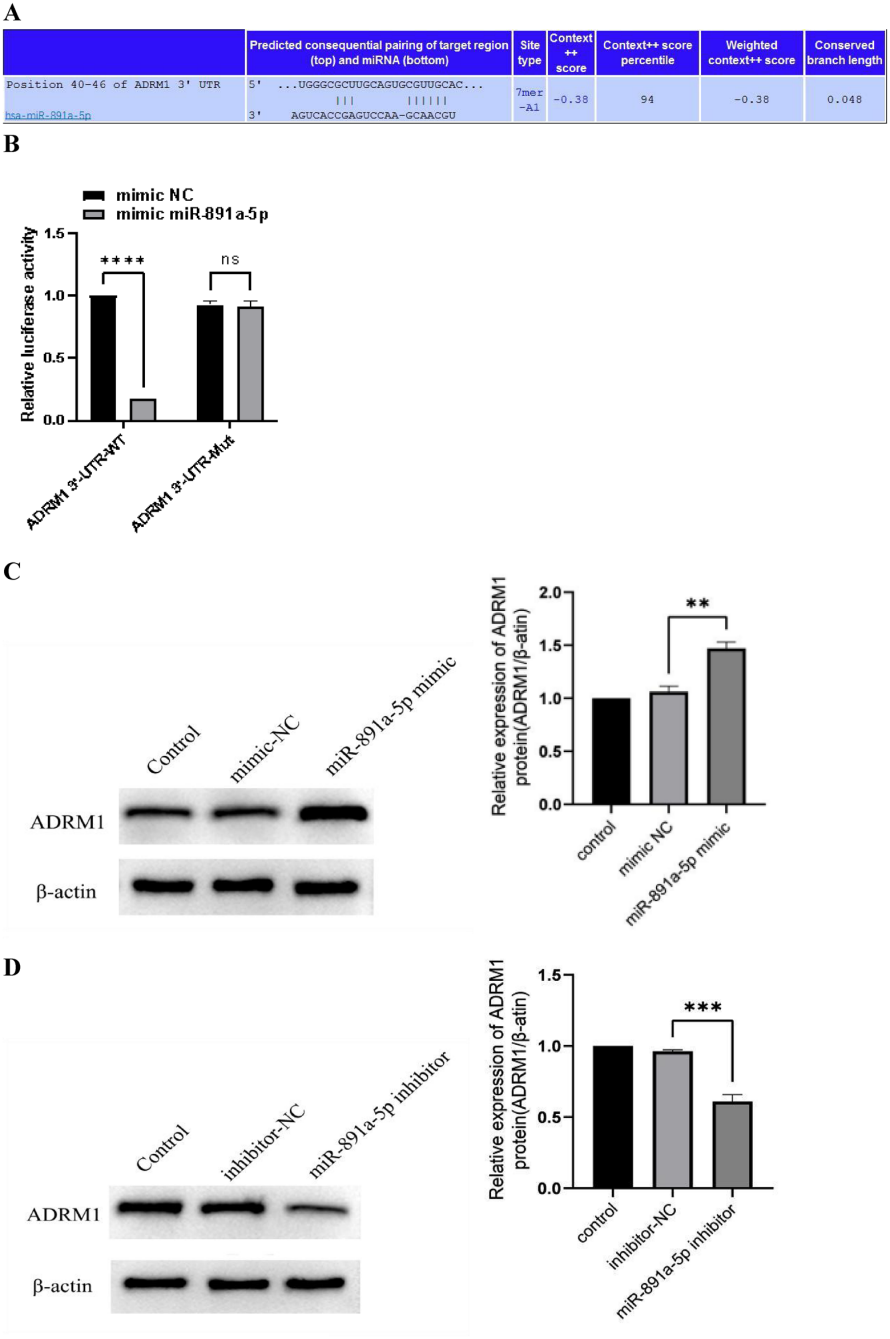
(**A**) Predicted binding site of miR-891a-5p on ADRM1 in the Starbase website. (**B**) The binding relationship between miR-891a-5p and ADRM1 identified using dual-luciferase reporter gene assay. (**C**, **D**) Western blotting for ADRM1 protein levels following miR-891a-5p mimic/inhibitor transfection. Original blots/gels are presented in Supplementary Figure. The samples derive from the same experiment and that gels/blots were processed in parallel.

First, we constructed the wild and mutant ADRM1-3′UTR into luciferase reporter vectors (Fig. 8A). It was verified that miR-891a-5p had a strong inhibitory effect on the wild-type 3′UTR of ADRM1 but had no significant effect on the mutant 3'UTR (Fig. 8B). It is proved that miR-891a-5p binds to ADRM1-3′UTR. RT-qPCR and western blotting were performed to observe the expression of ADRM1 in tumors. The overabundance of miR-891a-5p in Huh-7cells notably enhanced the levels of ADRM1 in terms of both mRNA and protein (Fig. 8C, *P* = 0.0016). In contrast, decreases in ADRM1 mRNA levels and protein levels were significantly downregulated when miR-891a-5p was down-regulated (Fig. 8D, *P* = 0.0001). These results provide convincing evidence that miR-891a-5p plays a positive role in regulating ADRM1 expression in LIHC by directly targeting the 3′-UTR region of ADRM1.

## Discussion

Hepatocellular carcinoma (HCC) is a common cancerous tumor of the gastrointestinal tract. Despite rapid advancements in diagnostic and treatment techniques for liver cancer, HCC remains a significant healthcare challenge because of its poor prognosis. The use of immune checkpoint inhibitors (ICI) has demonstrated effectiveness in treating HCC^18^. As a result, a significant amount of research has been conducted on prognostic indicators in HCC, aiming to discover well-established biomarkers that can effectively predict the response to ICI therapy; however, prognosis remains uncertain. This emphasizes the crucial requirement for developing a prognostic biomarker specifically customized for the immunotherapy of HCC. Recent studies have shown that ADRM1 plays crucial roles in the onset and progression of numerous human cancers. Nevertheless, there is a dearth of understanding regarding ADRM1 in HCC, necessitating further investigation.

Using R Software, we initially examined ADRM1 expression in various types of cancer. We performed a survival analysis of ADRM1 in the cancer types of interest. These findings indicate that elevated levels of ADRM1 are associated with an unfavorable prognosis in various cancers, including HCC patients. According to Yu-Cen Liang's research, ADRM1 is highly expressed in HCC, potentially contributing to its advancement of HCC^19^. Both this study and that conducted by Liang indicate that ADRM1 plays an oncogenic role in HCC.

Increasingly, research suggests that immune infiltration is crucial in the management of LIHC^20,21^. We found a significant correlation between ADRM1 and multiple immune cells such as B cells, CD4^+^T cells, Macrophages, Neutrophils, and Dendritic cells. Furthermore, ADRM1 expression was significantly positively correlated with biomarkers related to the presence of infiltrating immune cells. These findings suggest that tumor immune infiltration contributes to the oncogenic effects of ADRM1 in HCC.

The efficacy of immunotherapy depends on the invasion of tumor tissue and its effective detection. We validated the correlation between ADRM1 and immune cells in liver cancer cells using flow cytometry. Therefore, we will conduct a more in-depth examination of the correlation between ADRM1 and immune checkpoints. These findings indicate a strong association between the increased expression of ARDM1, PDCD1, CTLA4, and HAVCR2. Therefore, the discovery of these immune checkpoints and their correlation with ADRM1 will improve our understanding of the intricate interactions between immune regulation and the advancement of hepatocellular progression. Additional studies are required to clarify the fundamental processes and possible treatment implications of ADRM1 and its immune checkpoints in LIHC.

MIRNAs, which are approximately 22 nucleotides long^22^, are small RNA molecules that regulate gene expression by interacting with the mRNA of the target genes via binding. During the study, we predicted the following: By utilizing StarBase, it was found that miRNA891a-5p has the potential to target ADRM1. Subsequently, the validity of this prediction is confirmed using implementation of the dual-luciferase reporter assay. A subsequent investigation revealed that overexpression of miR-891a-5p greatly enhanced ADRM1 expression in Huh-7cells. miRNA-891a-5p has been demonstrated to be a new indicator that can potentially control the biological activities of tumor cells in breast, colorectal, and lung cancers^23–25^. The progression of liver cancer is regulated by a novel mechanism discovered in the current study involving miR-891a-5p/ADRM1 axis.

In summary, we found that ADRM1 is highly expressed in many tumors and is closely associated with the prognosis of patients with HCC. It has been reported that ADRM1 can promote tumor invasion and the expression of immune checkpoint proteins, thus promoting its tumor-promoting effects. Follow-up experiments revealed that ADRM1 participates in the occurrence and development of HCC, with miR-891a-5p/ADRM1 at its core. However, few basic and large-scale clinical studies are needed to confirm the results of this study.

## Materials and methods

### Individuals and biological specimens

Twenty patients with primary and precancerous tumors underwent liver surgery at the Second Affiliated Hospital of Xuzhou Medical University. All patients have received pathological verification. All the patients and their families provided informed consent. After the surgery, an appropriate amount of tissue samples was taken and immediately stored at − 80 °C for freezing, and performing RNA analysis at an appropriate time later.

### Cell cultures

10% fetal bovine serum (Palo Alto) and 1% penicillin (penicillin 100 U/mL) (penicillin 100 U/mL and streptomycin 100 ug/ml) were added to DEME (Palo Alto Gibco, California, USA), and the Huh-7 Cell lines (Manassas, VA, USA) was cultured in RPMI-1640 medium together with 1% penicillin (penicillin 100 U/mL) and streptomycin 100 ug/ml. The cells were stored at 37° in an incubator containing 5%CO_2_ (Thermo Science, Waltham, Massachusetts, USA) and changed periodically for 24 h to observe the growth state of the cells. Passage was performed using 0.25% trypsin every 72 h.

### Cell transfection

According to the manufacturer’s instructions, cells were transfected with negative control (NC)-mimic, ADRM1-mimic, negative control (NC)-inhibitor, and ADRM1 inhibitor using Lipofectamine 2000 reagent (Invitrogen, Carlsbad, CA, USA). In addition, cells were co-transfected with NC mimic and overexpression (OV)-NC plasmid, NC-mimic and OV-ADRM1 plasmid, miR-891a-5p-mimic and OV-NC plasmid, or miR-891a-5p-mimic and OV-ADRM1 plasmid.

### Quantitative real-time polymerase chain reaction (qRT-PCR)and reverse transcription

Total RNA was extracted from HCC tissues and cells using TRIzol reagent (Tiangen, Beijing, China). The cDNA was obtained by reverse transcription using an miRNA reverse transcription kit (Tiangen, Beijing, China). The reverse transcription primers and other primers for miR-891a-5p (F:5′-GTGCAACGAACCTGAGC-3′, R:5′-GACCTGAACCTGAACCTGAA-3′) and ADRM1 (F:5’AAGTACTTGGTGGAGTTTCG3’, R:5’ATGATCAAGTCGTC TTCCAC3’) were synthesized by Takara (Dalian, China). A reaction amplification system containing 1µl cDNA and 10µl was prepared using fluorescence quantitative PCR (Beijing, AQ101–02). Conditions: Pre denaturation at 94 °C for 5 min, denaturation at 94 °C for 25 s, annealing at 59 °C for 25 s, extension at 72 °C for 25 s, and finally extension at 72 °C for 5 min. ADRM1 to β-Actin(F:5′-GGCCCAGAATGCAGTTCGCCTT-3′R: 5′-AATGGCACCCTGC TCACGCA-3′) serves as an internal reference. Fluorescence quantitative PCR (ABI ViiA 7; Sun Yat-sen University Da An Gene Co., Ltd., Guangzhou, China). Quantitative PCR results were measured using 2^−∆CT^ technology. The experiment was repeated 4 times, and Thrice groups each time.

### Western blot

According to the recommended guidelines, the cells were removed and placed on a pre cooled 4 °C test bench using lysis buffer containing PMSF. A standard curve was plotted. Protein concentration was detected through colorimetric analysis using a biophotometer (Eppendorf). Proteins were separated using sodium dodecyl sulfate polypropylene (SDS-PAGE) and transferred to a nitrocellulose membrane (Millipore, Temecula, CA, USA). The film was sealed with skimmed milk powder at room temperature for 1 h. Using anti ADRM1 (Q16186, Millipore, Temecula, CA, USA), β-actin and incubate at room temperature for 2 h. The membrane was washed three times with three buffer solutions (TBS) containing 0.1% Tween-20 (TBST). They were then incubated with a secondary antibody, anti-rabbit immunoglobulin G (IgG) (R1131) at room temperature for 1 h. The immune response spectrum was determined using an enhanced chemiluminescence system (WBKLS0050). β-actin is the internal standard. A GIS500 gel image analysis system (Beijing Qianming Biotechnology Co., Ltd. Beijing, China) was used. Using the IMAGE J analysis method, the relative protein content was calculated based on the ratio of the target protein band grayscale to the internal reference protein band grayscale. Each experiment was conducted in triplicates.

### Assay for the reporter gene dual-luciferase

The Starbase database is used to analyze ADRM1 3′-Untranslated Region (UTR) and miR-891a-5p. In addition, the luciferase reporter gene experiment successfully established a correlation between ADRM13′-UTR and miR-891a-5p. ADRM1 was expressed in wild-type (wild-type) and mutant (Mut) cells using the dual-luciferase complex. Among these, miR-891a-5p formed complexes with ADRM1Wt, ADRM1Mut, and other proteins. MiR-891a-5p and dual-luciferase assay reporter genes were transferred into Huh-7liver cancer tissue. After 48 h, luciferase activity was measured using a luciferase analysis system (E1910, Promega, Madison, WI; USA). Activity was detected using the Reinella luciferase assay. Each experiment was conducted in triplicates.

### The CCK8 assay for cell counting

After washing twice with phosphate buffered saline (PBS), 0.25% trypsin was added to prepare a cell suspension. The suspension was added to a 96 well plate (6 × 10^6^ cells/well). Survival rate was measured using CCK8 test tubes (China Sangang Biotechnology Co., Ltd. E606335). Optical concentration (OD) was measured using an optical concentration meter (Nanjing Detai Testing Equipment Co., Ltd., China, BS-1101). Using the x-axis as time and the y-axis as the OD value, we plotted the changes in cell viability at each stage.

### Flow cytometric analysis

The cells were washed with PBS, and 0.25% trypsin was added to prepare a cell suspension. Add 20 µl of cell suspension and 20 µl of cell staining solution to a 6-well plate (operated on a 4 °C dark ice box). Transfer to flow cytometry after cell counting (1 × 10^6^). PBS was added and the mixture was centrifuged for 5 min before discarding the supernatant. Fluorescent monoclonal antibodies (CD3-FITC/CD16+56-PE/CD45-PerCP-Cy5.5/CD4-PC7/CD19-APC/CD8-APC-Cy7 Fluorescent monoclonal antibody, Tongsheng, Beijing, China) were used for 40 min and centrifuged for 10 min. Flow buffer (100 ml flow added, and data were collected on a Gallios flow cytometer (Beckman Coulter) for analysis using FlowJo software (Tree Star, San Carlos, California, USA).

### TCGA data download, process, and analysis

The mRNA expression data of 32 cancer types were downloaded from TCGA database (https://genome-cancer.ucsc.edu/), after which these data were normalized and then differential expression analysis was performed for ADRM1 using R package limma. Statistical significance was set at *P* < 0.05 significant.

### Analysis of the GEPIA database

GEPIA is an online high-throughput sequencing software (Genetic Transformation, GEPIA) based on TCGA and GTEx. GEPIA can be used to detect ADRM1 expression in many types of tumor cells. Simultaneously, the relationship between ADRM1, overall survival (OS), and disease-free survival (RFS) in 11 types of tumors (OS and RFS) was studied using GEPIA software. The logarithmic sequence method was used for the test, *P* < 0.05 was significant. GEPIA database was used to analyze the correlation between ADRM1 and immune test sites of liver cancer. *P* < 0.05 was used as the evaluation index, and a comparative analysis was carried out.

### Analysis of the TIMER database

The infiltrating immune cells of the tumor were detected using the Timer Web server (https://cistrome.shinyapps.io/timer/), to investigate the correlation between ADRM1 and tumor-infiltrating immune (*P* < 0.05).

### Analysis of the starBase database

starBase is a database containing both upstream and downstream genes for miRNAs. The purpose of this study was to confirm the relationship between miRNA-ADRM1 and the occurrence and development of liver cancer through the verification of clinical samples. A comparative study was carried out between hepatocellular carcinoma and healthy controls using STARBASE software. Furthermore, the miRNAs that interact with ADRM1 was screened by Starbase technique.

### RNA-sequencing expression

On this basis, the transcriptional groups of the three stages of ADRM1RNA were analyzed and compared with the clinical data of patients to determine the best normal state of ADRM1RNA. Using R-formula statistics, the *P* value and HR of each index were calculated and compared at 95% confidence intervals. Based on a multi-factor COX ratio risk analysis, the relationship between the total recurrence rate and the incidence of COX in X years was established. The nomogram was a curve relative to the recurrence rate of each patient in the RMSR toolbox. The results were analyzed using R software v4.0.3 and ggplot2 (v3.3.2). The results showed that there were significant differences between the two groups.

### Institutional review board statement

The study was conducted in accordance with the Declaration of Helsinki, and approved by Ethics Committee of the Second Affiliated Hospital of Xuzhou Medical University (protocol code [2022] 090501).” for studies involving humans.

### Informed consent

Informed consent was obtained from all subjects involved in the study.

### Supplementary Information

Supplementary Figures.

Supplementary Information.

## Data Availability

The researchers affirm that the information backing the discoveries of this investigation can be found in the article and its supplementary data.
