# Supplementary material for: The high expression of ADRM1 in hepatocellular carcinoma is closely related to tumor immune infiltration and is regulated by miR-891a-5p
Source: Sci Rep. 2024 Jun 18;14:14002. doi: 10.1038/s41598-024-64928-2 (PMC11189539; doi:10.1038/s41598-024-64928-2)
Supplement: Supplementary file 1 — Supplementary Figures. [file 41598_2024_64928_MOESM1_ESM.pdf]

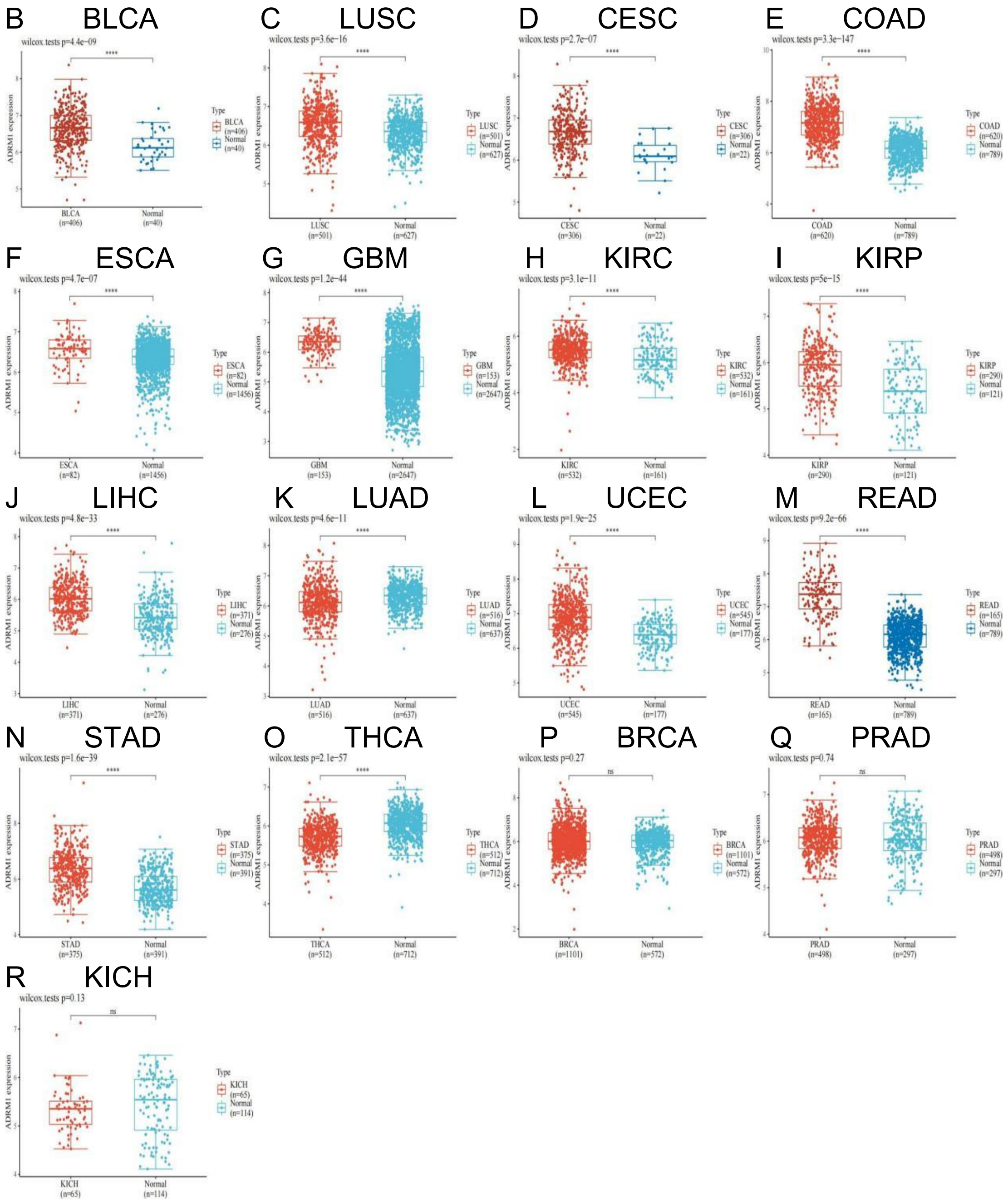

Figure S1. Comparison of ADRM1 expression in TCGA and normal tissues from TCGA and GTEx. *P<0.05,**P<0.01,***P<0.001.

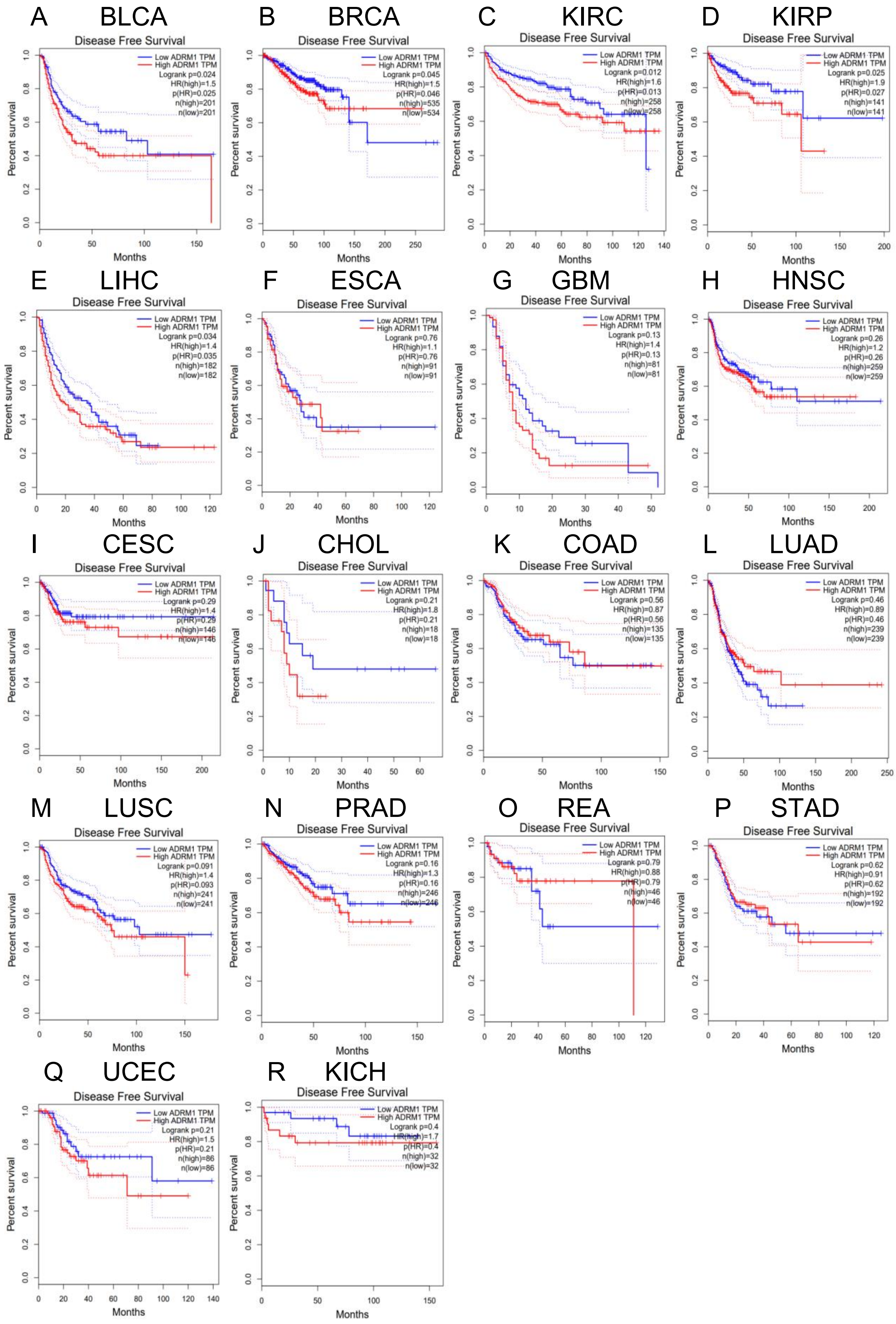

Figure S2. The GEPIA database analyzed the Disease-free survival (RFS) of ADRM1 in different types of human cancers. *P value <0.05.

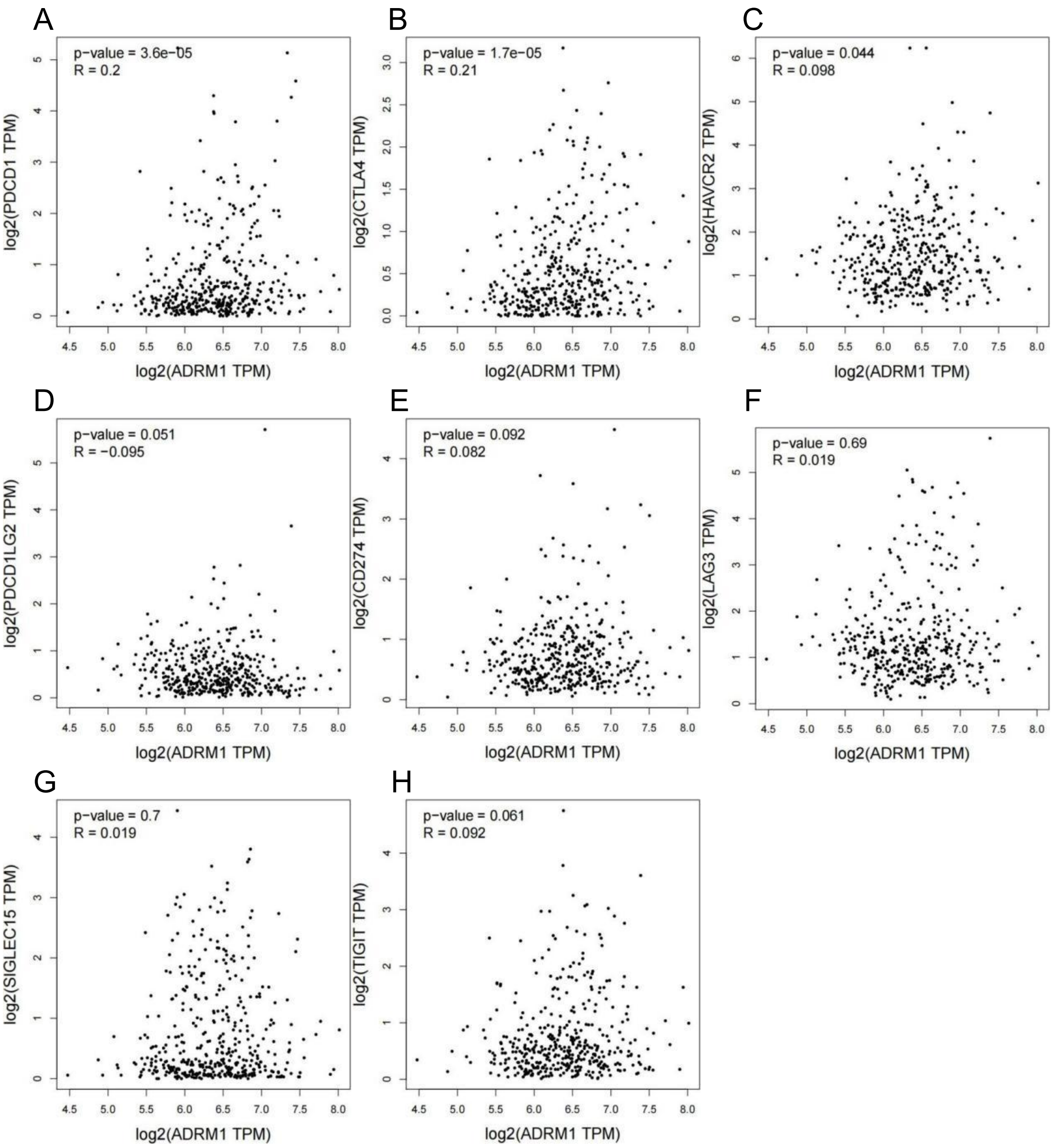

Figure S3. Correlation of ADRM1 expression with PDCD1, PDCD1LG2, CTLA4, CD274, HAVCR2, LAG3, SIGLEC15 and TIGIT expression in LIHC. A.The expression correlation of ADRM1 with PDCD1 in LIHC determined by GEPIA database. B. The expression correlation of ADRM1 with CTLA4 in HCC determined by GEPIA database. C. The expression correlation of ADRM1 with HAVCR2 in HCC determined by GEPIA database. D. The expression correlation of ADRM1 with PDCD1LG2 in LIHC determined by GEPIA database. E. The expression correlation of ADRM1 with CD274 in HCC determined by GEPIA database. F. The expression correlation of ADRM1 with LAG3 in HCC determined by GEPIA database. G. The

expression correlation of ADRM1 with SIGLEC15 in HCC determined by GEPIA database. H. The expression correlation of ADRM1 with TIGIT in HCC determined by GEPIA database.
