# Supplementary material for: The high expression of ADRM1 in hepatocellular carcinoma is closely related to tumor immune infiltration and is regulated by miR-891a-5p
Source: Sci Rep. 2024 Jun 18;14:14002. doi: 10.1038/s41598-024-64928-2 (PMC11189539; doi:10.1038/s41598-024-64928-2)
Supplement: Supplementary file 2 — Supplementary Information. [file 41598_2024_64928_MOESM2_ESM.pdf]

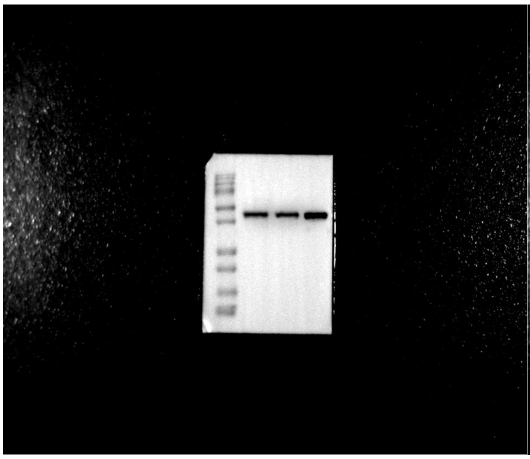

mir891a-5p mimic

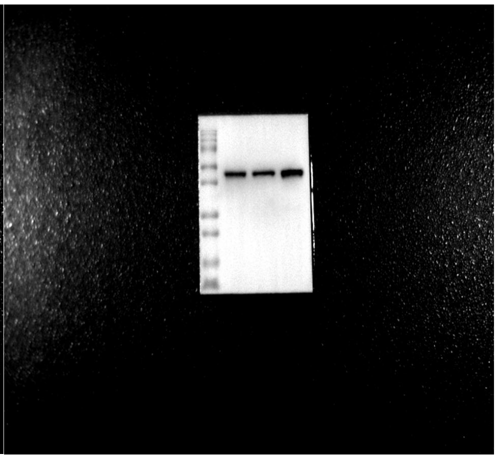

mir891a-5p mimic

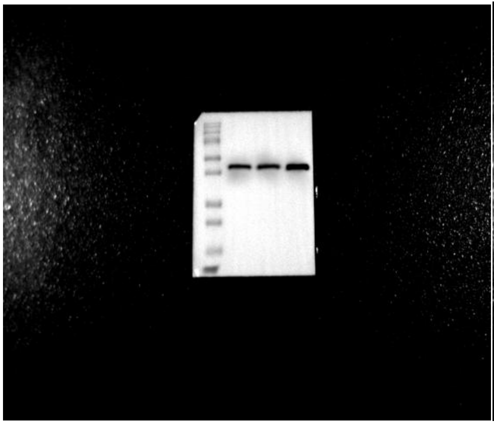

mir891a-5p mimic

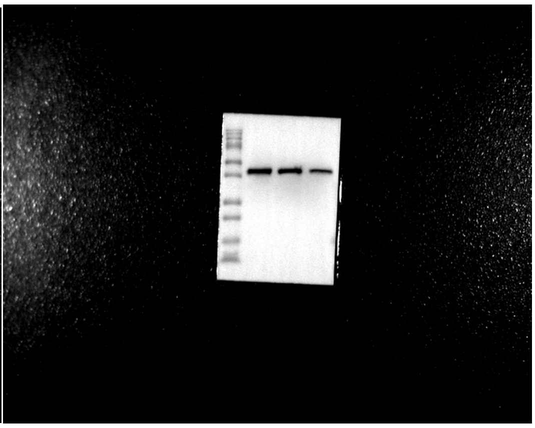

mir-8941a-5p inhibitor

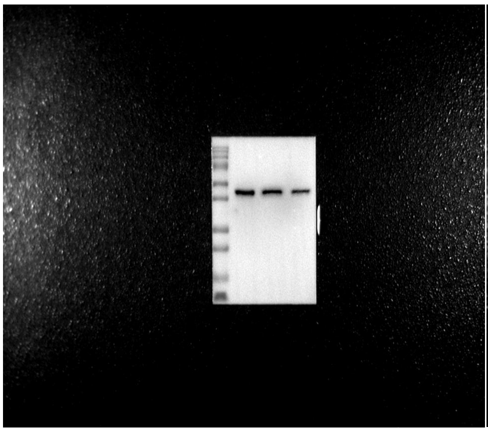

mir-8941a-5p inhibitor

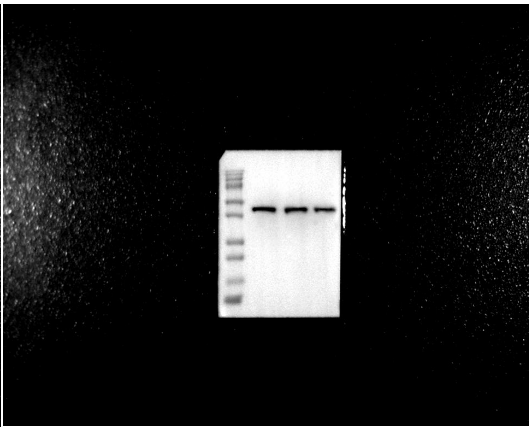

mir-8941a-5p inhibitor

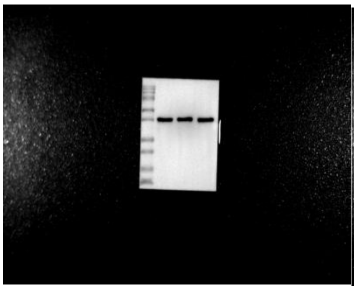

β -actin

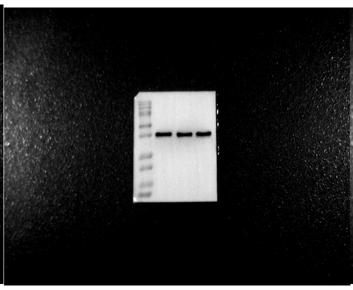

β -actin

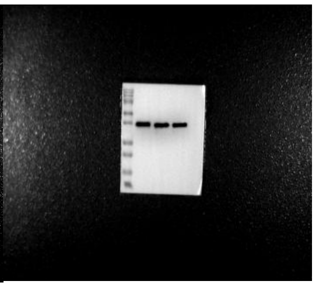

β -actin
